# Child Health Research Using linked Multi-domain Canadian Administrative Data: A Scoping Review

**DOI:** 10.23889/ijpds.v11i1.3407

**Published:** 2026-07-07

**Authors:** Yen My Vuu, Marcelo L. Urquia, Lisa M. Lix, Amani F. Hamad

**Affiliations:** 1 College of Community and Global Health, Rady Faculty of Health Sciences, University of Manitoba, Canada

**Keywords:** record linkage, parental linkage, population-based research, paediatric research, evidence synthesis

## Abstract

**Introduction:**

Linked administrative data integrating health and non-health information can support population-based research about biological and contextual environmental factors that influence child health. Database linkage studies leverage existing data to provide more comprehensive information than would be available from any single source. However, it is unknown the extent by which child health studies capitalise on linked multi-domain Canadian administrative data.

**Objective:**

This scoping review aims to describe Canadian population-based child health studies that used linked multi-domain (i.e., health and non-health) administrative data.

**Methods:**

A systematic search was conducted of MEDLINE, Embase, Scopus and Global Health from inception until March 12, 2025. Articles were included if they focused on children (birth to 18 years), used Canadian administrative data, and linked health with non-health data. Two reviewers independently screened titles/abstracts and full texts; a pilot test ensured consistency. Article characteristics, province/territory, parental linkage, and non-health variables, were collected using an extraction form.

**Results:**

The search yielded 4,437 articles, of which 42 met inclusion criteria. Most articles were conducted in Manitoba (45%) and Ontario (36%). Maternal linkage was common, whereas paternal linkage was limited to Manitoba and British Columbia. Immigration status was the most common non-health variable. Health service use, particularly preventive care, such as screening and vaccination coverage, was a common research theme. No multi-jurisdictional studies were identified.

**Conclusions:**

Multi-domain administrative data linkage studies remain concentrated in a few provinces. Expanding parental linkage, integrating non-health variables, and strengthening multi-jurisdictional studies are crucial for improving population-based understanding of child health influences across Canada.

## Introduction

Child health research helps uncover how biological and contextual environmental factors interact to influence child health outcomes. These factors are strongly interconnected; for example, socioeconomic adversity can negatively affect physical and mental health [1, 2], while compromised health in childhood may limit educational achievement [3]. Population-based studies allow researchers to capture these complex relationships through large-scale evidence [4]. Multi-domain administrative data offer a valuable source for studying child health, including physical, mental, and social well-being, by linking data on health and non-health domains [5–7].

Administrative data are routinely collected records to manage health care, education, social programs, and other sectors, rather than for research purposes; yet they capture large, representative populations that reflect real-world settings, making them valuable for research [4, 8, 9]. Population-based administrative data also offer key advantages as they are relatively low-cost, allow for long follow-up periods, and reduce challenges such as attrition that commonly affect primary data collection [10], which is often constrained by the need for parental consent, child participation, and long-term developmental follow-up [11–14]. Moreover, these barriers are compounded by imbalanced recruitment across socioeconomic and cultural groups, as well as loss to follow-up when families relocate, change schools, or transition between paediatric and adult care systems, all of which can lead to underrepresentation and biased results [15, 16]. Therefore, linked administrative data are useful in child health research by enabling long-term follow-up within large population-wide cohorts.

Linked multi-domain administrative data integrate information from different sectors (e.g., health, education, justice, social services) at the individual or family level [7]. They allow researchers to combine health datasets, such as physician claims, prescriptions, and immunisation records, with non-health datasets, such as education, child protection, and social services [4, 17]. In this review, a domain refers to a data sector representing a distinct aspect of people’s lives or service systems. For example, household income reflects socioeconomic status, typically obtained from provincial income support or social assistance records, while child protection involvement refers to contact with child welfare services. These non-health domains capture many of the social and structural determinants of health, such as poverty, family structure, and justice involvement, that influence children’s life trajectories but are often underrepresented in clinical data [18, 19]. Another key advantage of administrative data for child health research is the ability to link children’s records with those of parents and grandparents, enabling intergenerational analyses of family exposures and related child health risks [20, 21]. These advantages make administrative data particularly useful for studying child populations.

Canada’s universal systems and data infrastructures provide unique opportunities for linking health and non-health datasets for child health research [22]. Several Canadian provinces have established research data centres, such as the Manitoba Centre for Health Policy (MCHP), the Institute for Clinical Evaluative Sciences (ICES) in Ontario, and the Population Data BC (PopData) in British Columbia, which provide secure access to linked health and non-health datasets [23–25]. Despite Canada’s unique strengths in multi-domain administrative data, there is an unclear overview of how the data sources have been used to study children’s health. Examining how existing studies link health and non-health datasets is crucial to strengthen evidence on the influence of social, educational, and family contexts on child health outcomes and to identify future research opportunities. Although some reviews have examined how administrative data have been used to address specific topics, such as child maltreatment [26] or adult-focused mental health and addictions [27], no review has comprehensively summarised Canadian population-based child health studies that integrate both health and non-health domains from administrative data. Addressing this gap will help identify how health and non-health information are being linked across Canada and guide future use of administrative data in child health policy and research. Therefore, the purpose of this scoping review is to describe Canadian population-based child health studies that used linked multi-domain administrative data.

## Methods

### Protocol

This scoping review follows the Preferred Reporting Items for Systematic Reviews and Meta-Analyses extension for Scoping Reviews (PRISMA-ScR) reporting guidance [28] (https://www.prisma-statement.org/scoping), which outlines 20 essential and two optional items to promote consistent terminology and transparent reporting (Supplementary Appendix 1). A review protocol was not registered for this scoping review; however, the review followed PRISMA-ScR reporting guidance to ensure transparency and methodological rigour.

### Search Strategy

A search of the literature was conducted in MEDLINE, Embase, Scopus and Global Health from database inception (no publication date limits) until March 12, 2025. The search strategy was structured around three concepts: child population (birth to 18 years), administrative data, and Canada (Supplementary Appendix 2). Limiting the review to Canadian studies provided a context-specific description of multi-domain administrative data use in population-based child health research within one national framework. Including studies from multiple countries would introduce variability in healthcare systems and administrative processes, increasing heterogeneity in data types, linkage methodologies, and population characteristics. A Canadian focus, therefore, better supports national research planning, aligning with the goals of this review. Health domains covered a wide range of variables, such as birth outcomes, immunisations, and mental health. Non-health domains included social and demographic factors (e.g., income assistance, immigration status), education (e.g., grades, graduation), justice (e.g., charges, incarceration), and family services (e.g., foster care, welfare involvement). The environmental domain (e.g., pollution, climate change) was not included as a non-health domain because these data are not typically collected for service administration.

### Inclusion and Exclusion Criteria

Peer-reviewed journal articles were included if they met the following criteria: (1) focused on children from birth to 18 years, (2) were conducted using Canadian administrative data, and (3) included both health and non-health domains. Both single- and multi-jurisdictional studies were eligible. Articles were excluded if they met one or more of the following criteria: (1) methodological in scope (e.g., validation studies, methods papers, or protocol papers without sufficient information on linked administrative data sources, linkage approaches, and variables relevant to the objectives of this review), (2) did not include any health variables, (3) review articles, except as a tool to identify additional primary studies (4) conference abstracts, (5) non-population-representative cohorts, such as those restricted to children with specific clinical conditions (e.g., inflammatory bowel disease), or service involvement (e.g., child and family services care) to focus on research representing the general child population, (6) studies that used survey-derived measures (e.g., area-level income quintile), which are not routinely collected for administrative purposes.

### Selection Process

Following the search, all articles were exported to Covidence, a web-based platform used for removing duplicates and screening (https://www.covidence.org/). Titles/abstracts and full-text screening of all articles were independently performed by two reviewers (AFH and YMV). Discrepancies were resolved through discussion, and consensus was achieved between the two reviewers; no third reviewer was consulted. Two reviewers independently screened titles/abstracts and full texts, with pilot tests conducted before each screening stage to ensure consistency. A third pilot involving five random articles was later conducted before the full data extraction stage to test the extraction form. For each pilot, both reviewers independently screened the same set of articles.

Inter-reviewer agreement was evaluated using both the percentage of agreement and Cohen’s kappa (*κ*). The percentage of agreement measures the proportion of screening decisions for which reviewers selected the same articles based on the inclusion and exclusion criteria, while *κ* adjusts for agreement expected by chance to provide a robust estimate of inter-rater reliability [29], with 95% confidence intervals for both measures calculated using standard error estimates derived from the observed proportionate agreement and sample size. *κ* values were interpreted according to established benchmarks, with values indicating slight (< 0.20), fair (0.21-0.40), moderate (0.41-0.60), substantial (0.61-0.80), and almost perfect (>0.80) agreement [29].

### Data Extraction

An extraction form was developed to collect article characteristics. One reviewer (YMV) extracted information for the final analyses, resolving any uncertainties in consultation with a second reviewer (AFH). A pilot data extraction was conducted independently by the two reviewers to ensure consistency and accuracy in the extraction process. Extracted information included article title, study province/territory, publication year, parental linkage status, health variables, non-health variables, and data sources. Research themes were identified from article titles, which represent the primary focus of each article (Supplementary Appendix 3).

### Data Analysis

Descriptive analyses were subsequently conducted to summarise the distribution of studies by province, publication year, parental linkage, non-health variables, and research themes in counts and proportions. Inter-reviewer agreement through percentage of agreement and *κ* was calculated at the screening stage to evaluate consistency in article selection. Analyses were performed using R (version 4.4.2).

## Results

### Article Selection and Screening Agreement

**Figure 1:**
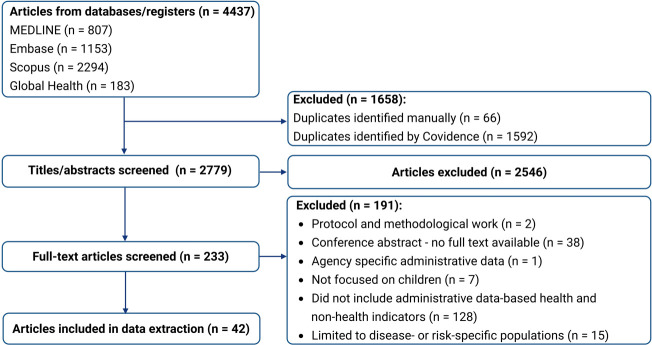
PRISMA Flow Diagram of Article Selection Process

The initial database search yielded 4,437 articles: MEDLINE (n = 807), Embase (n = 1,153), Scopus (n = 2,294), and Global Health (n = 183). After removing 1,658 duplicates, including 1,592 identified by Covidence and 66 manually, 2,779 articles remained for titles/abstracts screening. Of these, 2,546 articles were excluded for not meeting the inclusion criteria. Full-text screening was conducted on the remaining 233 articles, from which 42 met the eligibility criteria and were included in the final data extraction. Reasons for exclusion at the full-text stage were documented (Figure 1).

Inter-reviewer agreement was high across all stages. For the titles/abstracts screening stage, agreement in the pilot test was 78% (95% CI: 70-86%), while agreement overall was 95% (95% CI: 94-96%) with a *κ* of 0.65 (95% CI: 0.58-0.70). For the full-text screening stage, agreement in the pilot test was 80% (95% CI: 66-94%), while agreement overall was 92% (95% CI: 89-96%) with a *κ* of 0.75 (95% CI: 0.64-0.86). During data extraction, an agreement of 98% was reached in a pilot test.

### Article Characteristics

**Figure 2:**
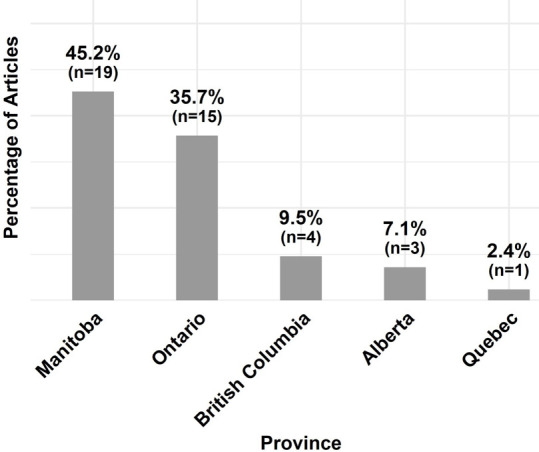
Frequency and Percentage of Articles by Province (n: number of articles)

**Figure 3:**
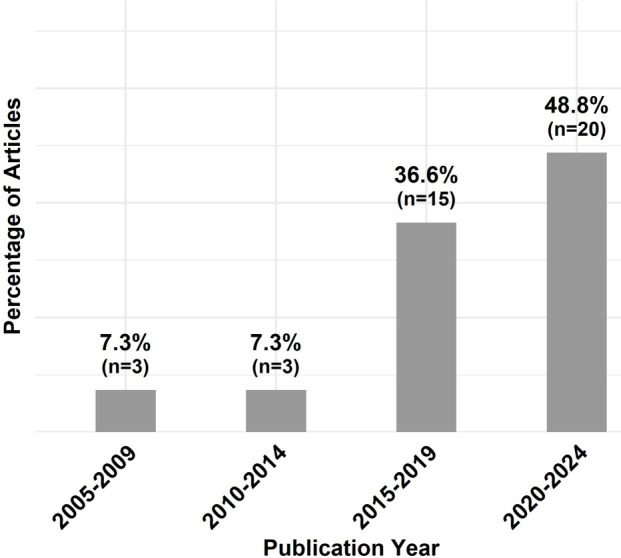
Frequency and Percentage of Articles by Publication Year (n: number of articles)

**Figure 4:**
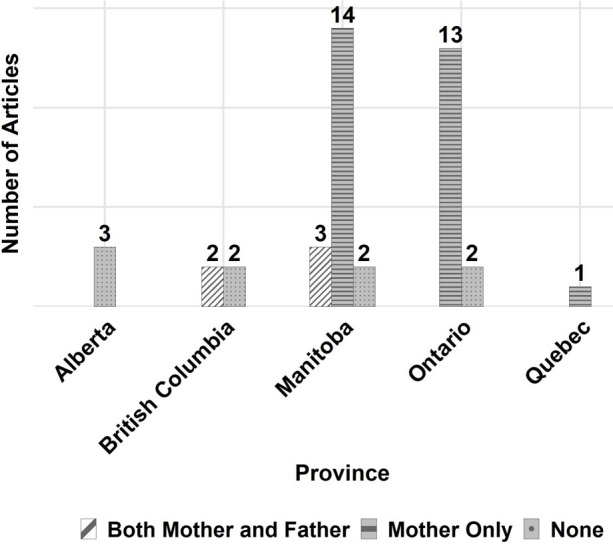
Frequencies of Articles by Parental Linkage and Province

All 42 selected articles were conducted within a single province or territory; no multi-jurisdictional studies were identified. Manitoba accounted for the highest percentage (45.2%, n = 19) of the total 42 articles, followed by Ontario (35.7%, n = 15) (Figure 2). The number of publications increased over time; nearly half (48.8%, n = 20) were published between 2020 and 2024 (Figure 3). Parental linkage varied across the provinces (Figure 4). Maternal linkage was most common, used either exclusively or in combination with paternal linkage. Only Manitoba and British Columbia linked to both parents; other provinces linked only to mothers or did not include parental linkage.

#### Non-health Variables

**Figure 5:**
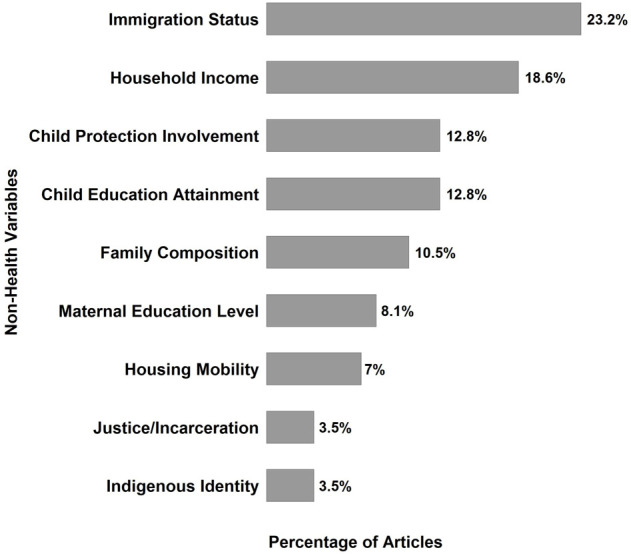
Reported Non-health Variables for Included Articles

The most frequently used non-health variables were immigration status (23.2%) and household income (18.6%), together accounting for approximately 40% of all reported non-health variables (Figure 5). Other non-health variables included child protection involvement (12.8%), child educational attainment (12.8%), and family composition (10.5%). Few studies included maternal education level, housing mobility, justice/incarceration, or Indigenous identity.

### Research Themes

The 42 articles encompassed five research themes, which reflected how linked administrative data have been applied to population-based child health research in Canada. Health service utilisation, particularly preventive care, was the most common theme, including studies on healthcare service use [1, 30–34], screening programs [35], vaccination coverage [36–39], and public health programs [40]. Maternal and infant health was the second common theme, focusing on maternal health [41, 42], prenatal exposures [43], birth outcomes [44–47], breastfeeding [48], and neurodevelopmental outcomes [49, 50]. The theme of child and adolescent mental health was also common, including studies on mental health status [51, 52], anxiety [53], psychotic disorders [54, 55], childhood maltreatment [56], and mental health care utilisation [1, 31, 32, 34]. Another common theme was the social and economic determinants of health, which reported income inequality through the lens of income support or social assistance [2, 46, 57–59], material and social circumstances such as food security [60] and parental immigration background influenced both child health outcomes and patterns of health service use [47, 55, 61]. Lastly, education and developmental outcomes examined the effects of health and social factors on school performance, long-term academic achievement [2, 3, 17, 58, 62–64] and early-life social influences on adolescent outcomes [64, 65].

## Discussion

This review identified 42 Canadian articles using multi-domain linked administrative data in population-based child health research, all conducted within a single province or territory, reflecting Canada’s decentralised data systems [66, 67]. This decentralised concentration may also reflect differences in data governance frameworks, availability of established data repositories, researcher capacity, and funding across provinces or territories. Similar systems exist in other countries, such as Australia and the United States, where multi-jurisdictional collaboration requires complex coordination [68, 69]. However, emerging strategies are beginning to facilitate coordinated multi-jurisdictional data access. Initiatives such as Health Data Research Network Canada (HDRN) aim to improve and coordinate access to administrative data across multiple jurisdictions [70]. Similar national data coordination exists internationally, such as Health Data Research UK (HDR UK) in the United Kingdom and the Population Health Research Network (PHRN) in Australia, which provide secure, federated infrastructures to support cross-jurisdictional data linkage [71, 72]. In situations where direct coordination is not feasible, meta-analysis offers an alternative approach by pooling region-specific estimates to generate overall national estimates while maintaining privacy protections that prevent the sharing of data between regions [73]. As a result, multi-jurisdictional research is necessary to improve generalizability in child health research findings.

Of the 42 included articles, nine did not include any parental linkage, and most linked only to mothers. This limitation constrains the ability to examine intergenerational risk factors and family level associations; for example, linking both parents allows for comprehensive analyses of how parental justice involvement and household income influence child health outcomes [74–76]. It also supports advanced analytical designs, including sibling and cousin comparisons, which can strengthen causal inference [77]. Expanding both-parent linkage is therefore essential to capture the full family context and advance robust intergenerational research on child health.

Across studies, the commonly reported non-health variables were immigration status and household income, while limited studies reported on information related to Indigenous identity and parental justice involvement. Studying child health requires linking health and non-health data, because child health outcomes are influenced by education, family, and social factors [4, 5]. Linking these datasets at the individual or family level allows exposures (e.g., poverty, child protection involvement) and outcomes (e.g., vaccination coverage, academic achievement) to be measured within the same population, particularly in vulnerable groups who may be underrepresented in health data alone [18, 19]. Other important non-health variables were largely absent or limited in the 42 included articles, such as housing instability and childhood adversities. This likely reflects how administrative data are generated and structured, as these variables are not routinely captured in administrative data and are often confined to sector-specific systems, such as social services, where access and linkage are more complex, limiting their inclusion in population-based research. These variables are essential for understanding inequities rooted in socioeconomic and cultural contexts, and their inclusion would allow researchers to examine disparities in child health outcomes to better support marginalised populations. Addressing this limitation will require better integration of data across sectors, more consistent collection of key social determinants, and transparent processes to ensure equitable data collection.

This review has strengths and limitations. First, it provides a comprehensive review of Canadian population-based child health studies using linked multi-domain administrative data. Second, the review followed PRISMA-ScR guidelines and applied predefined eligibility criteria to ensure transparency and reproducibility. Third, two reviewers independently screened all articles at the titles/abstracts and full-text stages, resolving disagreements through discussion to maintain review quality. Fourth, the search strategy was developed in collaboration with a health sciences librarian, strengthening its comprehensiveness through database-specific adaptations. However, the search strategy might not have captured all relevant articles, as some publications may have used alternative terminology (e.g., medical claims) that did not align with our searching strategy. In addition, this review did not search grey literature sources, such as government or institutional reports, which often use administrative data but are not published in peer-reviewed journals. Future studies could expand the current search strategy by incorporating additional generic or alternative terms and grey literature searches to optimise the retrieval of relevant articles.

## Conclusion

This scoping review highlights the growing use of multi-domain administrative data in Canadian population-based child health research. To advance the field, future research should strengthen multi-jurisdictional collaboration, enhance parental linkage and expand linkages to additional non-health domains to better capture the wide range of social determinants that shape child health outcomes and to produce comparable evidence that informs child health policy and services across Canada.

## Supplementary Appendices

Supplementary Appendix 1: The PRISMA-ScR checklist. Supplementary Appendix 2: Search strategy the databases. Supplementary Appendix 3: Information of 42 articles included in the scoping review.
